# Tracking temporal variations of fatality and symptomology correlated with COVID-19 dominant variants and vaccine effectiveness in the United States

**DOI:** 10.3389/fpubh.2024.1419886

**Published:** 2024-09-18

**Authors:** Shao Lin, Han Liu, Quan Qi, Ian Trees, Donghong Gao, Samantha Friedman, Xiaobo Romeiko Xue, David Lawrence

**Affiliations:** ^1^Department of Environmental Health Sciences, School of Public Health, University at Albany, State University of New York, Albany, NY, United States; ^2^Department of Epidemiology and Biostatistics, School of Public Health, University at Albany, State University of New York, Albany, NY, United States; ^3^Department of Sociology and Demography, University of Texas at San Antonio, San Antonio, Texas; ^4^Department of Economics, University at Albany, State University of New York, Albany, NY, United States; ^5^Epidemiology Branch, Division of Population Health Research, Eunice Kennedy Shriver National Institute of Child Health and Human Development, National Institutes of Health, Rockville, MD, United States; ^6^Department of Sociology, University at Albany, State University of New York, Albany, NY, United States; ^7^Wadsworth Center, New York State Department of Health, Albany, Albany, NY, United States

**Keywords:** COVID-19, deaths, severe symptoms, mild symptoms, dominant viral variants, vaccination, booster

## Abstract

**Introduction:**

We described how COVID-19 fatality and symptoms varied by dominant variant and vaccination in the US.

**Methods:**

Using the Restricted Access Dataset from the US CDC (1/1/2020–10/20/2022), we conducted a cross-sectional study assessing differences in COVID-19 deaths, severity indicators (hospitalization, ICU, pneumonia, abnormal X-ray, acute respiratory distress syndrome, mechanical ventilation) and 12 mild symptoms by dominant variant/vaccination periods using logistic regression after controlling for confounders.

**Results:**

We found the highest fatality during the dominant periods of Wild (4.6%) and Delta (3.4%). Most severe symptoms appeared when Delta was dominant (Rate range: 2.0–9.4%). Omicron was associated with higher mild symptoms than other variants. Vaccination showed consistent protection against death and severe symptoms for most variants (Risk Ratio range: 0.41–0.93). Boosters, especially the second, provided additional protection, reducing severe symptoms by over 50%.

**Discussion:**

This dataset may serve as a useful tool to monitor temporospatial changes of fatality and symptom for case management and surveillance.

## Introduction

While many studies have assessed the infection and death rates of specific COVID-19 variants, little research systematically compared the symptomatology of all viral variants and how vaccination affects COVID-related fatality rates and symptoms. COVID-19 has led to more than 103 million cases and over 1 million deaths in the US (1). On January 20, 2020, the first US COVID-19 case (Wild variant) was identified in Washington state (2, 3). A new variant, Alpha (B.1.1.7), emerged in December 2020 and became the dominant variant in March 2021 (2, 4). Three months later, the Delta variant (B.1.617.2) caused a sharp increase in COVID-19 cases, leading to the peak incidence in the nation. By the end of 2021, Omicron infection (B.1.1.529) was detected and subsequently led daily cases to surge to over a million, becoming yet another dominant variant that extended within 2 weeks (2). Therefore, it is critical to understand how COVID-19’s viral variants affected deaths and severe symptoms (5, 6).

The first FDA-approved vaccine against COVID-19 was released to curb viral infection and transmission in December 2020 (2). One month later, the second dose became widely available. To protect against severe diseases and death, the Centers for Disease Control and Prevention (CDC) recommended the first booster shot for individuals over 5 years old in September 2021 and authorized the second booster dose on March 29, 2022 (7). As of October 2022, 68.25% of Americans completed the primary series, including the first and second doses, while less than 50% finished the first booster dose (8). However, the effectiveness of vaccines against different SARS-CoV-2 variants, especially regarding fatality and specific symptoms, is still unknown.

Prior studies in different countries have examined variations in death, hospitalization, and ventilation rates concerning some variants and vaccines (9–16). For example, Bast et al. (17) found that hospitalization and ICU admissions related to the Delta variant were higher in the United States (U.S.) than the Alpha variant. Relative to the Delta variant, Omicron infection in the United Kingdom affected the lower respiratory tract less (10) and is associated with lower risks of ICU admissions, ventilation, and in-hospital mortality in France (9). Additionally, COVID-19 vaccines provide overall protection against risks of infection, hospitalization, and mortality, although the effectiveness depends on viral variant (6, 13, 14, 18). Furthermore, the protection from past COVID-19 infection was high (78%) against severe episodes (i.e., hospital admission or death) for 40 weeks for all variants except for Omicron (36.1%) (19).

In terms of research gaps, few studies compared fatality and symptomology across all variants, especially the new Omicron variant subtypes, and have found inconsistent results (10, 20). Past studies in the U.S. are also limited in generalizability due to using regional data or focusing on only one or two hospital systems (16, 19). Additionally, although multiple global studies have confirmed that vaccination can protect people against the adverse effects of COVID-19 (9, 13, 16, 21), its protective effects on various COVID-related symptoms by different viral variants are poorly understood.

This is the first exploratory study using the US nationwide surveillance data to (1) compare the differences in fatality and severe and mild clinical symptoms across all four COVID-19 dominant variants; (2) investigate the effectiveness of multiple vaccination doses and boosters’ availability against fatality and symptoms, and (3) assess the joint effects of COVID-19 viral variants and vaccinations on these outcomes.

## Methods

### Study design and controls

We used a cross-sectional design to compare clinical outcomes across different COVID-19 variants and vaccine availabilities individually and jointly. The “Alpha variant” was used as the control group in variant comparison because it was associated with the lowest fatality rate among all variants after controlling for sociodemographic characteristics and vaccine availability. The “No vaccine period” was used as the control group for the vaccine and joint effect analyses. Following prior research (22), we defined the time window of COVID-19 viral variants corresponding to a relative plateau for various viral subtypes and the population with each subtype within each window. A similar plateau concept was also used to define the time window of vaccination and boosters (i.e., considering the plateau of each vaccine and the population during that period). Specifically, we defined the variants plateau as below based on data reported by the Department of Health of all US states and territories (4), i.e., the original Wild variant from 1/20/2020 to 3/7/2021; the Alpha variant from 3/8/2021 to 6/13/2021; the Delta variant from 6/14/2021 to 12/12/2021; and Omicron since 12/13/2021. Meanwhile, the first vaccine in the US became available on 12/14/2020; the second dose on 1/14/2021; the first booster on 9/24/2021; and the second booster on 3/29/2022 (23).

### Data acquisition

All COVID-19 data in this study were obtained from the CDC Restricted Access Dataset. This dataset includes case status (confirmed or probable case), date of first positive specimen collection, demographics (sex, age group, race, ethnicity, and state of residence), pre-existing medical conditions (Yes/No without description), and the presence of mild and severe COVID-19 symptoms. The dates of symptom onset and first positive specimen collection were also reported. The CDC suppressed data cells reporting fewer than five records and combinations of uncommon demographic characteristics to prevent releasing personally identifiable data. The CDC surveillance system includes patient-level data reported by all US territories and states. Our analysis includes data from January 1, 2020 to October 20, 2022. We kept our analysis until October 2022 because the CDC changed its reporting data from daily to monthly after this date. In addition, COVID-19 self-testing kits became free and more available at this time, which may have led to substantial under-reporting of new cases from then on.

### Outcomes and predictors

The health outcomes in this study include death, 13 mild symptoms (fever, subjective fever, chills, myalgia, runny nose, sore throat, cough, dyspnea, nausea, vomiting, headache, abdominal pain, and diarrhea), and six severe symptoms (pneumonia, abnormal X-ray, ARDS, hospitalization, ICU, and MV/intubation). These symptoms are dichotomized variables (Yes/No). The fatality rate was calculated as the total number of deaths divided by the total number of cases multiplied by 100%.

### Statistical analysis and confounders

While the data of case symptom/death is at an individual level, all observations within each viral variant or vaccination time window are assigned to that corresponding variant/vaccination status. We used chi-square tests with Bonferroni correction to compare the rates of fatality and symptoms across dominant COVID-19 variants and vaccination stages. While rates were the major indicator for comparing symptomology by variants, we also developed logistic regression models by regressing each symptom predictor against periods of variant dominance/vaccine availability while controlling for sex, age group, race/ethnicity, lab confirmation status, state, season, and vaccination (for variant analysis) or variant (for vaccination analysis). These confounders were selected based on biological plausibility and previous literature (22). We then conducted sensitivity analyses to minimize biases and validate our findings. First, we repeated the main analyses using monthly vaccination coverage rates to refine this variable (instead of Yes/No). Second, we reran the original models controlling for two additional confounders: healthcare worker status and pre-existing medical conditions. These two variables were missing for more than 80% of the cases and were excluded in the main analysis. We used R 4.1.1 for all data cleaning and analysis.^1^

## Results

Our data include 90 million COVID-19 cases in the United States from January 2020 to October 2022. Among all COVID-19 cases, 46.3% occurred during the Omicron period, followed by Wild variants (31.2%), Delta (17.7%), and Alpha (4.8%).

Table 1 shows the total number of COVID-19 cases, numbers and fatality rates, and various symptoms across dominant COVID-19 periods. The fatality rate was highest for Wild variants (4.6%), followed by Delta (3.4%), Alpha (2%), and lowest in Omicron (1.5%). Furthermore, Delta had the highest rates for the most severe symptoms, including ICU (7.8%), pneumonia (6.6%), ARDS (2%), and MV/Intubation (2.4%) than other variants. Additionally, while hospital admission was a common indicator for most variants, we found that hospitalization rates decreased during the Omicron dominant period by approximately 1/3 compared to prior variants. On the other hand, Omicron was associated with the highest rates of most mild symptoms (14.1–84.8%). There were over 70% of the patients reported cough (84.8%), running nose (79%), headache (75.3%), and sore throat (73.4%) in the Omicron dominant period. The differences and trends described above remained robust after controlling for confounders (Supplementary Table S1).

**Table 1:** Case numbers and rates of COVID-19 deaths, severe and mild symptoms by dominant variants, US, January 2020 – October 2022.

	Wild variants	Alpha	Delta	Omicron	Chi-square test^a^
	(1/1/20–3/7/21)	(3/8/21–6/13/21)	(6/14/21–12/12/21)	(12/13/21–10/20/22)	
	*N* (%)	*N* (%)	*N* (%)	*N* (%)	
*N*	28,115,720	4,369,194	15,931,662	41,766,572	
**Death**	510,778 (4.6%)	37,098 (2.0%)	191,002 (3.4%)	174,419 (1.5%)	<0.001
Hospitalization	1,361,904 (10.0%)	180,653 (8.8%)	549,641 (8.8%)	683,543 (6.4%)	<0.001
ICU	109,451 (7.5%)	10,627 (6.6%)	38,808 (7.8%)	33,820 (7.5%)	<0.001
Severe symptoms					
Pneumonia	105,096 (5.7%)	11,614 (5.4%)	45,448 (6.6%)	23,866 (5.2%)	<0.001
Abnormal X-ray	70,273 (7.5%)	7,123 (7.1%)	28,181 (9.4%)	16,777 (9.9%)	<0.001
ARDS	28,950 (1.8%)	2,548 (1.3%)	11,657 (2.0%)	7,825 (1.8%)	<0.001
MV/Intubation	30,148 (2.3%)	2,830 (1.9%)	10,036 (2.4%)	7,439 (2.4%)	<0.001
Mild symptoms					
Fever	1,423,852 (37.4%)	155,732 (27.4%)	716,186 (36.9%)	640,497 (25.7%)	<0.001
Subjective fever	1,153,094 (48.2%)	167,368 (53.3%)	515,688 (56.0%)	510,601 (61.0%)	<0.001
Chills	1,560,033 (49.4%)	212,204 (53.8%)	681,083 (51.5%)	706,710 (61.5%)	<0.001
Myalgia	2,313,832 (62.7%)	301,475 (63.7%)	987,122 (61.4%)	954,004 (68.1%)	<0.001
Running nose	1,348,276 (56.8%)	146,217 (58.2%)	761,139 (66.0%)	845,636 (79.0%)	<0.001
Sore throat	1,569,472 (47.5%)	217,104 (50.2%)	773,126 (51.4%)	1,151,915 (73.4%)	<0.001
Cough	2,912,038 (70.8%)	390,546 (74.3%)	1,483,317 (77.2%)	1,646,007 (84.8%)	<0.001
Dyspnea	1,105,324 (35.2%)	133,238 (34.1%)	402,051 (30.6%)	346,300 (34.4%)	<0.001
Nausea/vomiting	604,711 (22.9%)	99,899 (28.1%)	317,717 (27.4%)	232,692 (27.6%)	<0.001
Headache	2,636,626 (68.4%)	347,431 (70.1%)	1,210,630 (69.9%)	1,181,802 (75.3%)	<0.001
Abdominal pain	265,247 (12.4%)	35,481 (13.5%)	114,550 (12.5%)	77,877 (14.1%)	<0.001
Diarrhea	1,063,578 (35.2%)	135,500 (35.0%)	422,543 (32.5%)	333,255 (33.8%)	<0.001

a Bonferroni correction with the number of outcomes evaluated.

Table 2 presents the effect of vaccination on the rates of death and symptoms. With increasing vaccination availability, the fatality rate continuously decreased from 5.1% (pre-vaccination) to 4.8% (first dose), 3.1% (second dose), 2.2% (first booster), and 1.0% (second booster). We also found that vaccination was significantly associated with reduced risks for all severe symptoms compared to no vaccination after controlling for confounders (Table 2). Specifically, the risks of all severe symptoms gradually and consistently reduced after each vaccination, from the first dose through the second booster. The second booster was associated with the greatest drop in severe symptoms, with RR ranging from 0.24 to 0.61 (*p* < 0.05 for all severe symptoms). Meanwhile, the mildest symptoms, such as chills, running nose, sore throat, nausea/vomiting, cough, and headache, increased after vaccination (RR range: 1.11–1.92), though fever consistently and significantly reduced after various vaccine doses (RR range: 0.68–0.88, all *p* < 0.05).

**Table 2:** Comparisons of COVID-19 deaths and symptoms over vaccine availability, US, January 2020 – October 2022.

	No vaccine		First dose		Second dose		First booster		Second booster	
	(1/1/20–12/13/20)		(12/14/20–1/3/21)		(1/4/21–9/23/21)		(9/24/21–3/28/22)		(3/29/22–10/20/22)	
	Deaths: 355,066		Deaths: 65,731		Deaths: 237,544		Deaths: 207,281		Deaths: 47,675	
	%	RR (95% CI)	%	RR^a^ (95% CI)	%	RR^a^ (95% CI)	%	RR^a^ (95% CI)	%	RR^a^ (95% CI)
**Death**	5.1%	1.00	4.8%	0.77 (0.76, 0.78)	3.1%	0.63 (0.62, 0.64)	2.2%	0.53 (0.52, 0.54)	1.0%	0.19 (0.19, 0.19)
Hospitalization	11.3%	1.00	8.1%	0.80 (0.79, 0.81)	8.4%	0.74 (0.73, 0.74)	7.0%	0.71 (0.70, 0.72)	6.5%	0.46 (0.45, 0.47)
ICU	7.5%	1.00	8.2%	1.00 (0.96, 1.03)	7.6%	0.90 (0.88, 0.93)	7.5%	0.97 (0.92, 1.01)	6.8%	0.61 (0.58, 0.63)
Severe symptoms										
Pneumonia	6.1%	1.00	5.1%	0.88 (0.86, 0.91)	5.6%	0.80 (0.77, 0.82)	6.5%	0.82 (0.79, 0.85)	3.5%	0.27 (0.25, 0.28)
Abnormal X-ray	8.0%	1.00	6.5%	0.90 (0.86, 0.94)	7.9%	0.86 (0.83, 0.90)	10.2%	0.95 (0.90, 1.01)	7.3%	0.33 (0.31, 0.35)
ARDS	1.9%	1.00	1.6%	0.82 (0.77, 0.87)	1.6%	0.76 (0.72, 0.80)	2.0%	0.78 (0.72, 0.84)	1.3%	0.24 (0.22, 0.26)
MV/Intubation	2.5%	1.00	1.9%	0.96 (0.89, 1.02)	2.2%	0.93 (0.87, 0.98)	2.6%	1.00 (0.92, 1.09)	1.6%	0.46 (0.42, 0.51)
Mild symptoms										
Fever	40.8%	1.00	33.3%	0.88 (0.87, 0.89)	34.6%	0.76 (0.75, 0.77)	25.7%	0.84 (0.82, 0.85)	31.0%	0.68 (0.67, 0.69)
Subjective fever	46.9%	1.00	50.3%	0.98 (0.97, 0.99)	53.5%	0.94 (0.93, 0.96)	56.5%	0.96 (0.94, 0.97)	66.3%	1.10 (1.08, 1.12)
Chills	47.7%	1.00	54.3%	1.08 (1.07, 1.09)	50.8%	1.09 (1.08, 1.11)	55.2%	1.13 (1.12, 1.15)	72.1%	1.40 (1.37, 1.42)
Myalgia	61.5%	1.00	67.4%	1.08 (1.07, 1.09)	61.8%	1.02 (1.01, 1.03)	63.7%	1.01 (1.00, 1.03)	75.9%	1.23 (1.21, 1.25)
Running nose	52.8%	1.00	67.0%	1.18 (1.16, 1.19)	61.4%	1.17 (1.16, 1.18)	72.4%	1.12 (1.10, 1.14)	89.4%	1.92 (1.89, 1.97)
Sore throat	45.9%	1.00	51.5%	1.10 (1.09, 1.11)	49.7%	1.14 (1.12, 1.15)	62.4%	1.15 (1.14, 1.17)	82.2%	1.88 (1.86, 1.91)
Cough	69.6%	1.00	75.1%	1.02 (1.01, 1.04)	73.4%	0.95 (0.94, 0.96)	80.4%	1.07 (1.06, 1.09)	91.6%	1.60 (1.58, 1.63)
Dyspnea	35.0%	1.00	38.2%	1.02 (1.01, 1.03)	32.3%	0.99 (0.98, 1.00)	31.1%	0.98 (0.96, 0.99)	41.3%	0.87 (0.85, 0.88)
Nausea/vomiting	22.1%	1.00	24.7%	1.10 (1.08, 1.12)	26.0%	1.12 (1.11, 1.13)	28.9%	1.09 (1.07, 1.11)	25.9%	1.11 (1.09, 1.14)
Headache	66.7%	1.00	73.2%	1.09 (1.08, 1.10)	69.4%	1.08 (1.07, 1.09)	71.8%	1.07 (1.06, 1.09)	81.6%	1.37 (1.35, 1.39)
Abdominal pain	13.0%	1.00	11.3%	1.02 (1.00, 1.04)	11.6%	0.96 (0.94, 0.97)	14.0%	0.93 (0.90, 0.95)	14.1%	0.94 (0.91, 0.96)
Diarrhea	34.4%	1.00	38.9%	1.10 (1.08, 1.11)	34.0%	1.03 (1.02, 1.05)	31.2%	0.97 (0.96, 0.99)	40.9%	1.02 (1.00, 1.04)

a Models control for dominant variant, sex, race/ethnicity, age, whether the case is lab-confirmed, state, and season; Reference group: No vaccine.

In the first sensitivity analysis with pre-existing medical conditions and health care worker status added into the model as additional confounders, the results for severe symptoms remain similar, but most mild symptoms significantly reduced (Supplementary Table S2). In another set of sensitivity tests using the monthly vaccination coverage rates (Supplementary Table S3), we found even stronger effects on severe symptoms, suggesting our main analysis might have underestimated the effectiveness of vaccines.

The joint effects of dominant variants and vaccination on COVID-19-related clinical outcomes are presented in Table 3 to separate viral variants’ effects from vaccinations. During the period of Wild variants, the first dose showed significant protective effects on fatality and most severe symptoms, except for ICU and MV/Intubation, but the second dose significantly reduced risks for fatality and all severe symptoms (RR range: 0.63–0.93, all *p* < 0.05). This protection against severe symptoms continued after Alpha became the dominant variant (RR range: 0.41–0.87, all *p* < 0.05) but decreased during the Delta period as shown by increased risks of fatality and severe symptoms (RR range: 1.09–2.12, all *p* < 0.05). Furthermore, the first booster significantly lowered Delta-associated fatality (RR_1st booster_ = 1.24 vs. RR_2nd dose_ = 1.48), though risks of severe symptoms remained high. The protective effects of boosters were also significant for Omicron variants. The second booster reduced risks for fatality and all severe symptoms during Omicron (RR range: 0.21–0.72, all *p* < 0.05). As for mild symptoms, we also observed protective effects of vaccination on fever associated with Wild variants and Alpha, but not for Delta and Omicron variants.

**Table 3:** Risk ratios of joint effects of dominant variants and vaccine availability on deaths and COVID-19 symptoms, US, January 2020 – October 2022.

	First dose (Wild variants)	Second dose (Wild variants)	Second dose (Alpha)	Second dose (Delta)	First booster (Delta)	First booster (Omicron)	Second booster (Omicron)
	(12/14/20–1/3/21)	(1/4/21–3/7/21)	(3/8/21–6/13/21)	(6/14/21–9/23/21)	(9/24/21–12/12/21)	(12/13/21–3/28/22)	(3/20/22–10/20/22)
	RR^a^ (95% CI)	RR^a^ (95% CI)	RR^a^ (95% CI)	RR^a^ (95% CI)	RR^a^ (95% CI)	RR^a^ (95% CI)	RR^a^ (95% CI)
**Death**	0.77	0.63	0.41	1.48	1.24	0.58	0.21
	(0.76, 0.78)	(0.62, 0.64)	(0.41, 0.42)	(1.46, 1.49)	(1.23, 1.26)	(0.57, 0.59)	(0.20, 0.21)
Hospitalization	0.80	0.74	0.68	1.09	1.05	0.73	0.47
	(0.79, 0.81)	(0.73, 0.74)	(0.67, 0.68)	(1.09, 1.10)	(1.04, 1.06)	(0.72, 0.73)	(0.47, 0.48)
ICU	1.00	0.90	0.87	1.15	1.22	0.91	0.57
	(0.96, 1.03)	(0.88, 0.93)	(0.85, 0.90)	(1.12, 1.17)	(1.19, 1.25)	(0.89, 0.94)	(0.56, 0.59)
Severe symptoms							
Pneumonia	0.88	0.80	0.79	1.66	1.71	1.10	0.36
	(0.86, 0.91)	(0.77, 0.82)	(0.77, 0.81)	(1.63, 1.69)	(1.67, 1.74)	(1.07, 1.13)	(0.35, 0.37)
Abnormal X-ray	0.90	0.86	0.84	2.03	2.23	1.68	0.59
	(0.86, 0.94)	(0.83, 0.90)	(0.81, 0.87)	(1.97, 2.08)	(2.16, 2.29)	(1.61, 1.74)	(0.57, 0.61)
ARDS	0.82	0.76	0.60	1.66	1.70	1.22	0.37
	(0.77, 0.87)	(0.72, 0.80)	(0.57, 0.63)	(1.60, 1.72)	(1.64, 1.77)	(1.17, 1.28)	(0.35, 0.39)
MV/Intubation	0.96	0.93	0.81	2.12	2.29	1.57	0.72
	(0.89, 1.02)	(0.87, 0.98)	(0.77, 0.86)	(2.04, 2.21)	(2.18, 2.39)	(1.48, 1.66)	(0.68, 0.77)
Mild symptoms							
Fever	0.88	0.76	0.71	1.21	1.33	1.22	0.99
	(0.87, 0.89)	(0.75, 0.77)	(0.70, 0.72)	(1.20, 1.22)	(1.32, 1.34)	(1.21, 1.23)	(0.98, 1.00)
Subjective fever	0.98	0.94	1.17	1.51	1.53	1.54	1.78
	(0.97, 0.99)	(0.93, 0.96)	(1.15, 1.18)	(1.50, 1.52)	(1.51, 1.54)	(1.52, 1.56)	(1.76, 1.80)
Chills	1.08	1.09	1.71	1.62	1.68	1.68	2.07
	(1.07, 1.09)	(1.08, 1.11)	(1.69, 1.73)	(1.61, 1.63)	(1.66, 1.69)	(1.66, 1.7)	(2.05, 2.09)
Myalgia	1.08	1.02	1.55	1.39	1.38	1.42	1.72
	(1.07, 1.09)	(1.01, 1.03)	(1.54, 1.57)	(1.38, 1.40)	(1.37, 1.39)	(1.41, 1.44)	(1.71, 1.74)
Running nose	1.18	1.17	2.13	2.12	2.02	2.26	3.88
	(1.16, 1.19)	(1.16, 1.18)	(2.10, 2.16)	(2.10, 2.13)	(2.00, 2.04)	(2.23, 2.29)	(3.83, 3.94)
Sore throat	1.10	1.14	1.72	1.52	1.54	3.01	4.92
	(1.09, 1.11)	(1.12, 1.15)	(1.70, 1.74)	(1.51, 1.53)	(1.53, 1.55)	(2.98, 3.04)	(4.87, 4.97)
Cough	1.02	0.95	1.41	1.87	2.11	2.12	3.17
	(1.01, 1.04)	(0.94, 0.96)	(1.40, 1.43)	(1.85, 1.88)	(2.09, 2.12)	(2.1, 2.14)	(3.14, 3.2)
Dyspnea	1.02	0.99	1.20	1.25	1.23	1.09	0.97
	(1.01, 1.03)	(0.98, 1.00)	(1.19, 1.22)	(1.24, 1.26)	(1.22, 1.24)	(1.08, 1.1)	(0.96, 0.98)
Nausea/Vomiting	1.10	1.12	1.84	1.56	1.52	1.48	1.51
	(1.08, 1.12)	(1.11, 1.13)	(1.82, 1.87)	(1.54, 1.57)	(1.50, 1.53)	(1.46, 1.5)	(1.5, 1.53)
Headache	1.09	1.08	1.63	1.46	1.45	1.46	1.86
	(1.08, 1.10)	(1.07, 1.09)	(1.61, 1.65)	(1.45, 1.47)	(1.43, 1.46)	(1.44, 1.47)	(1.84, 1.88)
Abdominal pain	1.02	0.96	1.22	1.20	1.16	1.11	1.12
	(1.00, 1.04)	(0.94, 0.97)	(1.20, 1.24)	(1.19, 1.22)	(1.15, 1.18)	(1.09, 1.13)	(1.10, 1.14)
Diarrhea	1.10	1.03	1.54	1.25	1.18	1.05	1.10
	(1.08, 1.11)	(1.02, 1.05)	(1.53, 1.56)	(1.24, 1.26)	(1.17, 1.19)	(1.03, 1.06)	(1.09, 1.11)

a Models control for sex, race/ethnicity, age, whether the case is lab-confirmed, state, and season; Reference group: no vaccine: 1/1/20–12/13/20.

Table 4 showed significant differences in fatality and symptoms by Omicron variants. While the highest fatality rate (1.9%) occurred during BA.1 period, fatality increased slightly from BA.2 (0.7%) to BA.5 (1.1%) over time. Other severe symptoms during the recent Omicron periods were still lower than those at BA.1 period. On the other hand, mild symptoms increased over time with different Omicron variants. The risk ratios of various symptoms during different Omicron periods compared to the BA.1 period were described in Table 5 significant coefficients highlighted in boldface. Consistently with Table 4 and while all severe symptoms reduced during later Omicron periods (BA.2, BA.2.12.1, and BA.5) compared to the BA.1 period, all mild symptoms significantly increased over the different Omicron periods.

**Table 4:** Case numbers and rates of COVID-19 deaths, severe and mild symptoms by omicron variants, US, January 2020 – October 2022.

	Omicron (BA.1)	Omicron (BA.2)	Omicron (BA.2.12.1)	Omicron (BA.5)	Chi-square test
	(12/13/21–3/20/22)	(3/21/22–5/15/22)	(5/16/22–6/19/22)	(6/20/22–10/20/22)	
	*N* (%)	*N* (%)	*N* (%)	*N* (%)	
*N*	26,908,388	3,034,308	3,435,838	7,532,097	
**Death**	141,480 (1.9%)	8,000 (0.7%)	8,829 (0.9%)	19,166 (1.1%)	<0.001
Hospitalization	436,367 (6.4%)	44,181 (4.6%)	50,158 (5.1%)	110,920 (6.5%)	<0.001
ICU	21,208 (7.8%)	1,668 (4.8%)	2,021 (5.2%)	4,557 (8.2%)	<0.001
Severe symptoms					
Pneumonia	16,695 (6.3%)	1,063 (2.4%)	1,308 (2.9%)	2,326 (3.8%)	<0.001
Abnormal X-ray	10,987 (11.8%)	773 (5.1%)	1,068 (5.5%)	1,941 (7.7%)	<0.001
ARDS	5,405 (2.2%)	346 (0.9%)	388 (0.9%)	804 (1.4%)	<0.001
MV/Intubation	5,260 (2.9%)	352 (1.2%)	341 (1.1%)	608 (1.5%)	<0.001
Mild symptoms					
Fever	351,077 (22.5%)	51,568 (28.1%)	67,941 (33%)	114,992 (35.3%)	<0.001
Subjective fever	256,416 (56%)	59,448 (65.2%)	56,141 (64%)	85,320 (70.3%)	<0.001
Chills	359,739 (53.8%)	76,451 (68.2%)	78,189 (71.3%)	121,695 (76.4%)	<0.001
Myalgia	510,375 (62.4%)	95,267 (71.1%)	99,409 (75.6%)	157,217 (80.1%)	<0.001
Running nose	433,703 (71.4%)	79,918 (85.5%)	104,069 (89.9%)	144,701 (91.6%)	<0.001
Sore throat	595,471 (66.8%)	128,766 (80.1%)	125,823 (82.1%)	191,653 (84.6%)	<0.001
Cough	841,029 (79.2%)	178,552 (89.8%)	186,849 (92.2%)	287,228 (92.8%)	<0.001
Dyspnea	189,276 (30%)	32,046 (35.1%)	35,072 (40.5%)	56,313 (47%)	<0.001
Nausea/vomiting	151,051 (27.5%)	17,361 (23.1%)	14,822 (22.9%)	23,642 (28.1%)	<0.001
Headache	634,652 (70.5%)	117,815 (77.6%)	124,091 (81.6%)	196,104 (85%)	<0.001
Abdominal pain	45,817 (13.4%)	6,386 (11.5%)	6,470 (12.9%)	9,088 (15.9%)	<0.001
Diarrhea	179,654 (29.3%)	30,693 (34.3%)	34,556 (40.8%)	56,086 (47.5%)	<0.001

^aBonferroni correction with the number of outcomes evaluated.^

**Table 5:** Comparisons of deaths and COVID-19 symptoms by dominant variants, US, January 2020 – October 2022.

	Omicron (BA.1)		Omicron (BA.2)		Omicron (BA.2.12.1)		Omicron (BA.5)	
	(12/13/21–3/20/22)		(3/21/22–5/15/22)		(5/16/22–6/19/22)		(6/20/22–10/20/22)	
	%	RR (Ref.)	%	RR^a^ (95% CI)	%	RR^a^ (95% CI)	%	RR^a^ (95% CI)
**Death**	1.9%	1.00	0.7%	0.58 (0.35, 0.97)	0.9%	0.58 (0.33, 1.01)	1.1%	0.61 (0.34, 1.1)
Hospitalization	6.4%	1.00	4.6%	0.91 (0.75, 1.09)	5.1%	0.91 (0.75, 1.12)	6.5%	1.13 (0.91, 1.41)
ICU	7.8%	1.00	4.8%	0.7 (0.42, 1.15)	5.2%	0.61 (0.35, 1.08)	8.2%	1.06 (0.57, 1.97)
Severe symptoms								
Pneumonia	6.3%	1.00	2.4%	0.77 (0.54, 1.1)	2.9%	0.68 (0.46, 1)	3.8%	0.81 (0.53, 1.23)
Abnormal X-ray	11.8%	1.00	5.1%	1.03 (0.68, 1.57)	5.5%	0.84 (0.53, 1.34)	7.7%	1.05 (0.64, 1.72)
ARDS	2.2%	1.00	0.9%	1.13 (0.63, 2.01)	0.9%	0.91 (0.47, 1.75)	1.4%	1.22 (0.61, 2.47)
MV/Intubation	2.9%	1.00	1.2%	0.49 (0.14, 1.7)	1.1%	0.42 (0.11, 1.65)	1.5%	0.54 (0.13, 2.25)
Mild symptoms								
Fever	22.5%	1.00	28.1%	1.07 (0.96, 1.19)	33%	**1.14 (1.01, 1.27)**	35.3%	**1.38 (1.23, 1.56)**
Subjective fever	56%	1.00	65.2%	0.96 (0.85, 1.09)	64%	0.98 (0.86, 1.12)	70.3%	**1.16 (1.02, 1.33)**
Chills	53.8%	1.00	68.2%	0.99 (0.88, 1.11)	71.3%	0.99 (0.88, 1.12)	76.4%	**1.24 (1.09, 1.4)**
Myalgia	62.4%	1.00	71.1%	0.99 (0.9, 1.1)	75.6%	1.01 (0.91, 1.12)	80.1%	**1.31 (1.18, 1.47)**
Running nose	71.4%	1.00	85.5%	0.98 (0.84, 1.15)	89.9%	0.94 (0.79, 1.11)	91.6%	1.03 (0.86, 1.22)
Sore throat	66.8%	1.00	80.1%	1.04 (0.94, 1.14)	82.1%	0.99 (0.9, 1.1)	84.6%	**1.18 (1.06, 1.32)**
Cough	79.2%	1.00	89.8%	1.08 (0.97, 1.2)	92.2%	**1.12 (1, 1.25)**	92.8%	**1.2 (1.06, 1.35)**
Dyspnea	30%	1.00	35.1%	0.88 (0.78, 1)	40.5%	0.93 (0.81, 1.07)	47%	**1.17 (1.02, 1.35)**
Nausea/Vomiting	27.5%	1.00	23.1%	0.89 (0.77, 1.02)	22.9%	1.01 (0.88, 1.17)	28.1%	**1.18 (1.02, 1.38)**
Headache	70.5%	1.00	77.6%	0.95 (0.86, 1.05)	81.6%	0.99 (0.89, 1.1)	85%	**1.29 (1.15, 1.44)**
Abdominal pain	13.4%	1.00	11.5%	0.92 (0.76, 1.1)	12.9%	1 (0.82, 1.21)	15.9%	**1.24 (1.01, 1.52)**
Diarrhea	29.3%	1.00	34.3%	0.85 (0.75, 0.97)	40.8%	0.99 (0.86, 1.13)	47.5%	**1.2 (1.04, 1.38)**

a Models control for vaccine availability, sex, race/ethnicity, age, whether the case is lab-confirmed, state, and season; Reference group: Omicron (BA.1).

## Discussion

### COVID-19 fatality and symptoms by dominant viral variants

In this study, Wild and Delta variants had the highest fatality rates, followed by Alpha and Omicron in the US. This pattern is consistent with prior studies. For example, Lauring et al. (6), using data from 21 hospitals across the US, found that the Delta variant had a higher in-hospital mortality rate (12.2%) compared to Alpha (7.6%) and Omicron (7.1%). Similarly, Bouzid et al.’s study on patients visiting emergency departments in Paris indicated that the in-hospital mortality rate decreased from 9.5% during Delta to 4.1% during Omicron (9). Because these prior studies mainly focused on severe patients, the fatality rates reported in their studies are higher than that from our data, but the differences across variants are consistent across studies. The low fatality associated with Omicron we found was also confirmed by Wang et al. (24) based on COVID-19 infection and death data from 50 countries, which found that the case fatality rate decreased from Delta to Omicron in 47 of them, though the specific magnitude of change differed across countries. Kim et al. (25) recently found that the Omicron variant had a lower fatality rate than the Delta variant.

In addition, our study also indicated that the rates of almost all severe symptoms were highest during the Delta-dominated period, two-to-four times higher compared to other viral variants. This finding is also consistent with prior studies. According to a review by Rashedi et al. (26), the risk of hospitalization during Delta was about two times higher than the Wild and Alpha variants. Lauring et al.’s (6) study on hospitalized patients in US hospitals also found higher rates of MV among patients infected by Delta (22.0%) than those infected by Omicron (14.9%) or Alpha (6.8%). Other studies focusing on the two recent variants, Delta and Omicron, also found similar patterns. For example, Bouzid et al.’s (9) study on Paris emergency department visits and Wrenn et al.’s (27) study on patients in a Nashville, Tennessee hospital system found reductions in MV from Delta to Omicron. Kim et al.’s study (28) on a small number of isolated COVID-19 cases in South Korea also confirmed few patients infected by Omicron needed supplemental oxygen.

Our results also revealed the variation in mild symptoms across COVID-19 variants, as they became more prevalent during the Omicron period. The newest Omicron variant showed significantly higher rates of most mild symptoms, such as cough, running nose, headache, and sore throat, than other variants. Similar to our findings, a study in the United Kingdom (2021) also suggested that runny nose, headache, and fatigue are common symptoms of Omicron (20). However, Menni et al. (10) suggested that Omicron is also associated with less prevalence of fever and headaches than Delta. These discrepancies across studies may be attributed to the continuous mutation of the virus.

It is important to consider the confounding effect of the COVID-19 seropositivity or past infection that may also play a critical role in protecting against subsequent infections. For instance, the entire population was seronegative during the Wild variant infection and then most people were still seronegative during the Delta wave. However, there was significant seropositivity during the Omicron and subsequent variant infections due to prior infection and vaccinations. A meta-analysis using 65 studies from 19 different countries found that the protection against severe disease from the past infection was high for all variants, with 90·2% for all variants except for Omicron BA.1 (88.9%) at 40 weeks (19). Therefore, the reductions in COVID-19 fatality and severe symptoms overtime could partially contribute to the immunity gained by past infection in population (18, 19). Unfortunately, we could not control for this confounder due to lack of reinfection data or individual vaccination data. However, our findings are similar as the findings from other cohort studies which had individual vaccination and reinfection data.

Precise functions of mutations associated with each variant have yet to be thoroughly investigated scientifically. Existing research has been mostly focused on mutations that can explain the replication efficiency, transmissibility, and immune evasion of the Delta and Omicron variants (29). Regarding disease severity and fatality, according to Rashedi et al.’s (25) review, mutations in the Delta variant make it more invasive in lung and colon cells. During the Delta dominant period, these mutations may have increased risks of severe symptoms, such as pneumonia and fatality. Importantly, we know little about the new Omicron variant’s potential mechanisms that lead to its unique symptomology.

### Impacts of vaccination affecting COVID severity

Our results indicated the strong protection of vaccinations against COVID-19 fatality. Specifically, the fatality rate reduced from 5.1% (pre-vaccination) to 4.8% (after the first dose) to 3.1% (after the second dose). The two boosters led to further reductions in the fatality numbers—a 56.9% decrease after the first booster and an 80.4% decrease after the second booster compared to the pre-vaccination period. These significant reductions in fatality confirm the protection of the vaccine and boosters found in prior studies. The efficacy of the mRNA COVID-19 vaccine was first reported by Polack et al. in late 2020 (30). A review by Marfe et al. (31) indicated that other types of vaccines (e.g., adenoviral vector) can also reduce fatality.

We also found that the risks of all severe symptoms dropped significantly by 7–47% after the first and second doses and 39–81% after the two booster doses, compared to the original unvaccinated level. The substantial reduction in severe outcomes associated with the second booster is probably due to its target population. The second booster mainly targeted the most vulnerable population (older adults and those with chronic or existing diseases, such as cancer, chronic kidney disease, asthma, etc.) during most periods under study (23). Consistent with our findings, a study covering more than 1 million persons in Israel found that boosters could reduce severe illness as soon as 12 days after inoculation (32). A recent global data review by Nka et al. (33) found that vaccination is key for effectively controlling the COVID-19 pandemic.

Our study also showed that vaccination provides limited protection against mild COVID symptoms and is only effective for a few mild symptoms like fever. In 2020, when no COVID-19 vaccine was available, Kaslow predicted protection against lower-level viral replication (i.e., early mild symptoms) may be more difficult to achieve than to protect against higher-level viral loads (i.e., more severe symptoms) that occur much later during infection (34). However, this theoretical prediction has rarely been examined systematically in empirical research, given that most prior studies focused on deaths and severe symptoms. Our study provided unique evidence supporting this hypothesis.

We found that the emergence of new variants may modify the effectiveness of vaccines. Specifically, vaccines can reduce fatality and severe outcomes for all viral variants except for Delta. Interestingly, the first booster may reduce fatality and some symptoms compared to the second dose during the Delta period. Therefore, our results do not necessarily suggest that vaccination is ineffective against the Delta variant, as it can still protect against death with a full vaccination regimen as recommended by the US CDC. According to Lauring et al.’s (6) study that compared 4,000 hospitalized patients infected by Delta, the vaccinated group experienced significantly lower risks of MV (14.5% vs. 24.8%) or ICU admission (30.7% vs. 43.0%) compared to the unvaccinated group, though slightly higher rates of in-hospital mortality (13.2% vs. 11.8%). Wang et al.’s analysis of country-level vaccine coverage and fatality also indicated that percentages of the country’s population receiving the first, second, and booster doses correlate with reductions in fatality even during Delta (24).

### Strengths and limitations

This study has several strengths. First, by using nationwide CDC surveillance data of 86 million cases and 0.9 million deaths, we minimized reporting bias, which is typically a major concern in survey research and studies based on media reports. Also, our findings represent the entire population in the US. Second, we comprehensively analyzed both severe and mild clinical outcomes, which is the first effort in this area. Third, the time frame of our study includes the most recent Omicron variant (up to October 2022) and vaccination efforts (second booster). Fourth, our analysis controlled for important confounders, including sociodemographic factors, dominant variants, and vaccination periods.

Some limitations should be acknowledged. Our data do not include individual-level vaccination status or variant determinations. Instead, we used the dominant variant and vaccine availability periods as proxy population indicators with an ecological study design. Therefore, our findings should be interpreted cautiously. As a result of this ecological fallacy, potential misclassifications of viral variants and vaccination status may occur, which may have led to non-differential bias and underestimated the real associations. Nevertheless, our results are consistent with smaller-scale studies that used precise individual-level data after controlling for vaccination status and viral variants in the US (6, 26) and around the world (9, 27, 35). Another limitation is that we treated each infection independently and cannot separate first-time infections from reinfections. Given that immunity developed during previous infections may have some protective effects in subsequent infections (15), we might have underestimated the severity of later variants, such as Delta and Omicron, because reinfections could drive down the rates of severe symptoms. However, given that data from the New York State indicated that COVID-19 reinfections only account for about 6% of total infections (36), this misclassification does not likely lead to considerable bias in our results. We will confirm our findings using unique personal identifiers to separate infection and reinfection in future research.

Furthermore, any discrepancies between state reporting systems or the change in testing rate (not included in CDC data) may have introduced non-differential biases and underestimated the associations found. In addition, confounding due to changes in exposure and behavior patterns (e.g., declining mask usage), demographics, and at-risk populations over time may also confound our estimates. These biases will be addressed in our future studies. Finally, the CDC surveillance data are a passive registry and have considerable missing values. Symptoms missing % across time are consistently which led the bias toward null. Our sensitivity analyses suggest that without controlling for the additional confounders with high missing rate, we might have underestimated our results.

## Public health implications

Our findings may have important public health implications if confirmed by additional studies. For government and researchers, this study suggested that the CDC Restricted Access Dataset may be a useful surveillance tool to continuously monitor temporal–spatial changes of fatality and symptomology of COVID-19. In addition, the modification effect of viral variants and the confounding effect of past infection in studying the association between vaccination and fatality/symptom severity should be considered. For health care systems, this study provided important information of symptomology by different variants and identified the urgent care demand for each COVID-19 variant or subtype which could help clinical facilities to plan and prepare hospital beds, diagnosis and treatment facilities, and medical staff. For the society and public, our results showed strong and consistent protective effects of vaccination and boosters against death and severe symptoms, even when the dominant variant has multiple mutations (e.g., Delta and Omicron). According to the CDC, only 33.5% of the population in the US have received the first booster dose by October 2022 (8). In the backdrop of emerging new variants (37) and waning protection of the original two-dose regimen, it may be important for all eligible persons to remain up to date with recommended vaccinations to best protect against COVID-19 associated severe symptoms, hospitalization, ED visits and death.

## Conclusion

Fatality and symptoms differed across COVID-19 variants. The Delta variant had the highest fatality and severe symptom rates, followed by the Wild variants Alpha and Omicron. However, Omicron had higher risks of mild symptoms than all three prior variants. Vaccination provided strong and consistent protection against fatality and severe symptoms for most variants, and boosters provided additional protection.

## Data Availability

The original contributions presented in the study are included in the article/Supplementary material, further inquiries can be directed to the corresponding author.

## Footnotes

^1^ https://www.r-project.org/
