# Supplementary material for: Tracking temporal variations of fatality and symptomology correlated with COVID-19 dominant variants and vaccine effectiveness in the United States
Source: Front Public Health. 2024 Sep 18;12:1419886. doi: 10.3389/fpubh.2024.1419886 (PMC11445176; doi:10.3389/fpubh.2024.1419886)
Supplement: Supplementary file 1 [file Table_1.docx]

**Supplementary tables**

**Table S1: Comparisons of deaths and COVID-19 symptoms by dominant variants, US, January 2020 – October 2022**

|  | **Wild Variants** | | **Alpha** | | **Delta** | | **Omicron** | |
| --- | --- | --- | --- | --- | --- | --- | --- | --- |
|  | (1/1/20–3/7/21) | | (3/8/21-6/13/21) | | (6/14/21-12/12/21) | | (12/13/21-10/20/22) | |
|  | % | RR^a^ (95% CI) | % | RR (Ref.) | % | RR^a^ (95% CI) | % | RR^a^ (95% CI) |
|  |  |  |  |  |  |  |  |  |
| **Death** | 4.6% | 1.52(1.49,1.55) | 2.0% | 1.00 | 3.4% | 3.57(3.51,3.63) | 1.5% | 1.67(1.63,1.71) |
|  |  |  |  |  |  |  |  |  |
| **Severe symptoms** |  |  |  |  |  |  |  |  |
| Hospitalization | 10.0% | 1.09(1.08,1.10) | 8.8% | 1.00 | 8.8% | 1.62(1.60,1.63) | 6.4% | 1.12(1.11,1.13) |
| ICU | 7.5% | 1.04(1.00,1.08) | 6.6% | 1.00 | 7.8% | 1.32(1.27,1.36) | 7.5% | 0.98(0.94,1.03) |
| Pneumonia | 5.7% | 1.01(0.97,1.04) | 5.4% | 1.00 | 6.6% | 2.10(2.03,2.17) | 5.2% | 1.35(1.30,1.41) |
| Abnormal X-ray | 7.5% | 1.03(0.98,1.08) | 7.1% | 1.00 | 9.4% | 2.41(2.31,2.52) | 9.9% | 1.82(1.71,1.93) |
| ARDS | 1.8% | 1.26(1.18,1.35) | 1.3% | 1.00 | 2.0% | 2.76(2.60,2.93) | 1.8% | 1.98(1.83,2.14) |
| MV/Intubation | 2.3% | 1.14(1.06,1.23) | 1.9% | 1.00 | 2.4% | 2.62(2.45,2.79) | 2.4% | 1.79(1.64,1.96) |
|  |  |  |  |  |  |  |  |  |
| **Mild symptoms** |  |  |  |  |  |  |  |  |
| Fever | 37.4% | 1.07(1.06,1.09) | 27.4% | 1.00 | 36.9% | 1.71(1.68,1.73) | 25.7% | 1.57(1.54,1.59) |
| Subjective fever | 48.2% | 0.81(0.80,0.82) | 53.3% | 1.00 | 56.0% | 1.29(1.28,1.31) | 61.0% | 1.30(1.28,1.33) |
| Chills | 49.4% | 0.64(0.63,0.65) | 53.8% | 1.00 | 51.5% | 0.95(0.94,0.96) | 61.5% | 0.95(0.93,0.97) |
| Myalgia | 62.7% | 0.66(0.65,0.67) | 63.7% | 1.00 | 61.4% | 0.90(0.89,0.91) | 68.1% | 0.92(0.91,0.94) |
| Running nose | 56.8% | 0.55(0.54,0.56) | 58.2% | 1.00 | 66.0% | 0.99(0.98,1.01) | 79.0% | 1.11(1.08,1.13) |
| Sore throat | 47.5% | 0.66(0.65,0.67) | 50.2% | 1.00 | 51.4% | 0.88(0.87,0.89) | 73.4% | 1.72(1.70,1.75) |
| Cough | 70.8% | 0.67(0.67,0.68) | 74.3% | 1.00 | 77.2% | 1.32(1.31,1.34) | 84.8% | 1.33(1.31,1.35) |
| Dyspnea | 35.2% | 0.82(0.81,0.84) | 34.1% | 1.00 | 30.6% | 1.04(1.03,1.05) | 34.4% | 0.92(0.90,0.94) |
| Nausea/Vomiting | 22.9% | 0.61(0.60,0.62) | 28.1% | 1.00 | 27.4% | 0.84(0.83,0.86) | 27.6% | 0.83(0.81,0.84) |
| Headache | 68.4% | 0.66(0.65,0.67) | 70.1% | 1.00 | 69.9% | 0.89(0.88,0.91) | 75.3% | 0.90(0.89,0.92) |
| Abdominal pain | 12.4% | 0.78(0.77,0.80) | 13.5% | 1.00 | 12.5% | 0.98(0.96,1.00) | 14.1% | 0.94(0.91,0.96) |
| Diarrhea | 35.2% | 0.67(0.66,0.68) | 35.0% | 1.00 | 32.5% | 0.81(0.80,0.82) | 33.8% | 0.72(0.71,0.73) |

Note: a. Models control for vaccine availability, sex, race/ethnicity, age, whether the case is lab-confirmed, state, and season; Reference group: Alpha variant.

**Table S2: Comparisons of COVID-19 deaths and symptoms by vaccine availability after controlling for additional confounders (HCW and comorbidity), US, January 2020 – October 2022**

|  | **First dose** | **Second dose** | **First booster** | **Second booster** |
| --- | --- | --- | --- | --- |
|  | RR^a^ (95% CI) | RR^a^ (95% CI) | RR^a^ (95% CI) | RR^a^ (95% CI) |
| **Severe symptoms** |  |  |  |  |
| Death | 0.79 (0.75, 0.83) | 0.62 (0.59, 0.65) | 0.46 (0.42, 0.49) | 0.09 (0.08, 0.10) |
| Hospitalization | 0.84 (0.82, 0.87) | 0.84 (0.82, 0.86) | 0.81 (0.78, 0.84) | 0.41 (0.39, 0.42) |
| ICU | 0.78 (0.73, 0.84) | 0.67 (0.63, 0.71) | 0.78 (0.71, 0.86) | 0.23 (0.21, 0.26) |
| Pneumonia | 0.91 (0.88, 0.95) | 0.80 (0.78, 0.83) | 0.82 (0.78, 0.87) | 0.22 (0.20, 0.23) |
| Abnormal X-ray | 0.90 (0.85, 0.95) | 0.80 (0.76, 0.84) | 0.80 (0.75, 0.86) | 0.25 (0.23, 0.28) |
| ARDS | 0.84 (0.77, 0.9) | 0.73 (0.68, 0.78) | 0.75 (0.68, 0.83) | 0.22 (0.19, 0.25) |
| MV/Intubation | 0.80 (0.71, 0.89) | 0.55 (0.50, 0.61) | 0.60 (0.52, 0.69) | 0.11 (0.09, 0.13) |
| **Other symptoms** |  |  |  |  |
| Fever | 0.90 (0.88, 0.91) | 0.77 (0.76, 0.78) | 0.84 (0.82, 0.85) | 0.59 (0.58, 0.61) |
| Subjective fever | 0.91 (0.9, 0.93) | 0.84 (0.83, 0.86) | 0.88 (0.86, 0.90) | 0.84 (0.82, 0.86) |
| Chills | 0.97 (0.96, 0.99) | 0.94 (0.93, 0.96) | 0.96 (0.94, 0.98) | 0.98 (0.96, 1.01) |
| Myalgia | 1.00 (0.98, 1.01) | 0.93 (0.92, 0.94) | 0.92 (0.90, 0.94) | 0.92 (0.91, 0.94) |
| Running nose | 1.16 (1.14, 1.18) | 1.20 (1.18, 1.22) | 1.06 (1.03, 1.08) | 1.87 (1.82, 1.92) |
| Sore throat | 1.00 (0.99, 1.02) | 1.02 (1.00, 1.03) | 1.03 (1.01, 1.05) | 1.42 (1.40, 1.45) |
| Cough | 0.98 (0.97, 0.99) | 0.90 (0.88, 0.91) | 0.98 (0.96, 1.00) | 1.23 (1.20, 1.26) |
| Dyspnea | 0.92 (0.90, 0.93) | 0.85 (0.84, 0.86) | 0.88 (0.86, 0.90) | 0.65 (0.63, 0.67) |
| Nausea/Vomiting | 0.98 (0.96, 1.00) | 0.93 (0.92, 0.95) | 0.92 (0.90, 0.94) | 0.78 (0.76, 0.80) |
| Headache | 1.01 (1.00, 1.03) | 1.00 (0.99, 1.02) | 0.97 (0.95, 0.99) | 1.05 (1.03, 1.08) |
| Abdominal pain | 0.94 (0.92, 0.97) | 0.87 (0.85, 0.89) | 0.84 (0.81, 0.87) | 0.70 (0.68, 0.73) |
| Diarrhea | 0.98 (0.96, 1.00) | 0.88 (0.87, 0.89) | 0.85 (0.83, 0.86) | 0.75 (0.74, 0.77) |

Note: a. Models control for dominant variant, sex, race/ethnicity, age, pre-existing medical conditions, whether the case is lab-confirmed, whether the case is a health care worker, state, and season; Reference group: No vaccine.

**Table S3: Comparisons of COVID-19 symptoms over monthly vaccination rates, US, January 2020 – October 2022**

|  | **First and second doses** | **First booster** | **Second booster** |
| --- | --- | --- | --- |
|  | RR^a^ (95% CI) | RR^a^ (95% CI) | RR^a^ (95% CI) |
|  |  |  |  |
| Death | 0.16 (0.15, 0.17) | 0.28 (0.27, 0.29) | 0.29 (0.29, 0.3) |
|  |  |  |  |
| **Severe symptoms** |  |  |  |
| Hospitalization | 0.88 (0.85, 0.9) | 0.51 (0.5, 0.52) | 0.74 (0.73, 0.75) |
| ICU | 0.83 (0.74, 0.93) | 0.61 (0.57, 0.66) | 0.67 (0.63, 0.71) |
| Pneumonia | 0.92 (0.83, 1.02) | 0.33 (0.31, 0.35) | 0.28 (0.26, 0.3) |
| Abnormal X-ray | 1.24 (1.07, 1.43) | 0.37 (0.34, 0.4) | 0.31 (0.29, 0.34) |
| ARDS | 0.51 (0.42, 0.62) | 0.3 (0.26, 0.33) | 0.26 (0.23, 0.29) |
| MV/Intubation | 0.88 (0.7, 1.09) | 0.45 (0.39, 0.52) | 0.6 (0.53, 0.68) |
|  |  |  |  |
| **Mild symptoms** |  |  |  |
| Fever | 0.99 (0.95, 1.03) | 0.87 (0.85, 0.89) | 0.82 (0.8, 0.84) |
| Subjective fever | 1.23 (1.18, 1.29) | 1.14 (1.11, 1.17) | 1.53 (1.49, 1.56) |
| Chills | 1.28 (1.23, 1.33) | 1.35 (1.31, 1.38) | 1.59 (1.56, 1.63) |
| Myalgia | 1.1 (1.07, 1.15) | 1.22 (1.19, 1.24) | 1.54 (1.51, 1.57) |
| Running nose | 1.43 (1.37, 1.5) | 1.58 (1.54, 1.63) | 2.24 (2.18, 2.31) |
| Sore throat | 1.76 (1.7, 1.83) | 1.97 (1.92, 2.01) | 1.86 (1.83, 1.9) |
| Cough | 1.89 (1.82, 1.96) | 1.59 (1.56, 1.63) | 1.74 (1.7, 1.78) |
| Dyspnea | 1.19 (1.14, 1.24) | 0.9 (0.87, 0.92) | 1.08 (1.06, 1.11) |
| Nausea/Vomiting | 1.4 (1.34, 1.46) | 1.04 (1.01, 1.07) | 1.26 (1.23, 1.3) |
| Headache | 1.15 (1.11, 1.2) | 1.31 (1.28, 1.34) | 1.57 (1.54, 1.6) |
| Abdominal pain | 1.12 (1.06, 1.19) | 1.08 (1.04, 1.12) | 1.16 (1.12, 1.2) |
| Diarrhea | 1.02 (0.98, 1.06) | 0.99 (0.97, 1.02) | 1.23 (1.2, 1.26) |

Note: a. Models control for dominant variant, coverage rates of prior doses, sex, race/ethnicity, age, whether the case is lab-confirmed, state, and season; Effect sizes multiplied by the coverage rates in October 2022 (68.25% for the first and second doses, 33.5% for the first booster, and 7.9% for the second booster).
